# Cross-cultural associations between behavioural, emotional, and cognitive differences in autistic children and parental wellbeing: evidence from five countries

**DOI:** 10.3389/fpsyt.2026.1850089

**Published:** 2026-07-17

**Authors:** James Rufus John, William Suwandi Budiman, Christa Lam-Cassettari, Poppy Zenzi Grimes, Kiran Hingorani, Ying Qi Kang, Ramkumar Aishworiya, Erdembileg Sundarimaa, Marta Volgyesi-Molnar, Krisztina Stefanik, Agota Szekeres, Ileana Mardare, Florina Rad, Valsamma Eapen

**Affiliations:** 1 School of Clinical Medicine, University of New South Wales, Sydney, NSW, Australia; 2 Academic Unit of Infant, Child, and Adolescent Psychiatry Services, South Western Sydney Local Health District, Liverpool, NSW, Australia; 3 Ingham Institute of Applied Medical Research, Liverpool, NSW, Australia; 4 Swalcliffe Park School CIO, Oxfordshire, United Kingdom; 5 Yong Loo Lin School of Medicine, National University of Singapore, Singapore, Singapore; 6 Child Development Unit, Khoo Teck Puat – National University Children’s Medical Institute, National University Hospital, Singapore, Singapore; 7 Hungarian Academy of Sciences - ELTE University 'Autism in Education' Research Group, Budapest, Hungary; 8 Faculty of Special Education, Institute of Special Needs Education for People with Atypical Behaviour and Cognition, ELTE University, Budapest, Hungary; 9 Hungarian University of Agriculture and Life Sciences, Institute of Education, Kaposvár, Hungary; 10 University of Medicine and Pharmacy “Carol Davila”, Bucharest, Romania

**Keywords:** autism spectrum disorder, autistic difficulties, parental stress, quality of life, transcultural

## Abstract

**Introduction:**

Parental quality of life (QoL) and stress are strongly linked to behavioural, emotional, and cognitive differences in autistic children. However, these relationships have rarely been examined with a cross-cultural lens, despite the potential influence of sociocultural contexts on assessment and interpretation. To address this knowledge gap, this study employed multinational data to investigate the cross-cultural associations between emotional/behavioural and cognitive/adaptive differences in autistic children and parental wellbeing.

**Methods:**

Data were obtained from 219 autistic children aged up to 18 years across five countries (Australia, Singapore, Hungary, Romania, and the United Kingdom). Standardised instruments were used to assess four key domains of parental QoL, parental stress, child’s behavioural/emotional difficulties, and adaptive/cognitive differences. Since different standardised instruments were used across countries, all measures were dichotomised according to established clinical cut-offs for each instrument. Multivariable logistic regression models were used to examine associations between children’s autistic difficulties and parental wellbeing accounting for country- and measure-level variations.

**Results:**

Across countries, children experiencing higher emotional/behavioural difficulties were significantly associated with lower parental QoL (QoLA Part A: AOR = 0.36, 95% CI 0.11-0.97) and twice higher odds of parental stress (AOR = 2.11, 95% CI 1.08-4.54). However, the association between cognitive/adaptive difficulties and parental QoL and parental stress did not reach statistical significance. Additionally, there were no evidence of country- or measure-level heterogeneity for parental wellbeing outcomes (ICC = 0.00), indicating consistent associations across cultural contexts and assessment tools.

**Discussion:**

Emotional and behavioural difficulties in autistic children are significantly associated with poorer parental quality of life and increased stress across diverse cultures, suggesting that the challenges faced by parents are largely universal. These findings highlight the importance of early identification and family-centred, culturally responsive supports to improve wellbeing for both autistic children and their parents.

## 1 Introduction

Autism spectrum disorder (ASD, hereafter autism) is a neurodevelopmental disorder with varying levels of impact across behavioural, emotional, cognition, and adaptive domains (1), with global prevalence estimates ranging widely from 1.09 to 436 per 10,000 people (2). The neurodevelopmental differences in autism are often exacerbated by other comorbidities, with studies reporting around one-third to half of autistic individuals experiencing a co-occurring psychiatric condition (2, 3). Further, children with cognitive issues are 2.5 to 4 times more likely to exhibit behavioral difficulties (3) and up to ten times more likely to develop comorbid psychiatric conditions (4, 5) compared to those without such issues. Autistic children with comorbid psychiatric conditions often experience complex support needs, and may benefit from respite care for caretakers (6) and the involvement of larger healthcare teams for comprehensive care (7). Differences in cognitive (thinking and reasoning) and adaptive (practical) skills can further compound behavioural (e.g., hyperactivity, restricted behaviours) and emotional (e.g., empathy, social communication) difficulties, underscoring the interdependence of these developmental domains (8, 9). These interrelated difficulties highlight the wide-ranging impact on both children and their families.

There is substantial evidence on the impact on parental and caregiver wellbeing with reports of higher levels of stress compared to those without autism or children with other developmental conditions (10, 11). Stressors can arise from managing emotional and behavioural issues (3, 6), necessitating complex navigation of specialised educational systems (12, 13), and coping with societal stigma (14). Furthermore, autism also imposes substantial economic costs for affected individuals and their families with the need for intensive and sustained support services, reduced parental workforce participation, and the need for specialised interventions (15–17). Further, parental quality of life (QoL) encompassing physical health, emotional wellbeing, social functioning, and overall life satisfaction may be affected, with evidence showing that higher severity of child behavioural and emotional difficulties as consistently linked to increased parental stress and reduced QoL, highlighting the interdependence between child functioning and parental wellbeing (14, 18).

Although the relationship between parental wellbeing and difficulties associated with caring for autistic children is well-documented, much of the research has been conducted in single-country or culturally homogeneous samples. Evidence on how autistic child’s emotional/behavioural and cognitive/adaptive differences relate to parental wellbeing across different cultural contexts is limited but crucial, as cultural norms, parenting styles and practices, healthcare systems, and societal expectations may influence both the child and parental experiences (6, 19, 20). Cross-country comparisons can illuminate whether associations between child difficulties and parental outcomes are consistent globally, or whether cultural and systemic factors moderate these relationships (21). To address this knowledge gap, this study aimed to investigate the cross-cultural associations between emotional/behavioural and cognitive/adaptive differences in autistic children and parental wellbeing, specifically stress and QoL across five countries: Australia, Singapore, UK, Romania, and Hungary. By leveraging data from diverse cultural contexts (i.e., Western, Asian, Eastern and Central European cultures), this study provided a unique opportunity to explore the generalisability of these associations and to inform culturally sensitive interventions and supports for families of autistic children.

## 2 Methods

### 2.1 Study design and sites

This study employed a cross-sectional, observational design using multinational data collected from parents of autistic children across five countries: Australia, Singapore, the United Kingdom (UK), Romania, and Hungary. Data were collected as part of ongoing national and regional research projects on autism, with ethical approval obtained in each participating country according to local regulations. The cross-country design allowed for examination of both within-country associations and cross-cultural differences in parental wellbeing in relation to autistic children’s differences.

### 2.2 Study participants

Parents or primary caregivers of autistic children in each country were recruited. Participants were recruited through ongoing national and regional autism research projects in clinical services, educational settings, or community networks within each participating country using locally approved recruitment procedures. As this was a collaborative multinational study utilising existing datasets, sample sizes varied across countries, with Australia contributing a comparatively larger proportion of participants than the other sites. Inclusion criteria were: (1) child aged 3 to 18 years, (2) confirmed autism diagnosis via clinical assessment or standardised diagnostic criteria, and (3) availability of parent-reported measures of wellbeing (i.e., either stress or QoL or both).

### 2.3 Study measures

Given the cross-country nature of this collaboration, standardised instruments were used wherever possible to ensure comparability across sites. All measures were administered in their validated English-language versions across participating countries, except in Hungary and Romania, where validated translated versions of the instruments were used. However, due to variations in available tools and research protocols, measures assessing child emotional/behavioural difficulties, cognitive/adaptive functioning, and parental stress differed across countries. To enable harmonisation for pooled analyses, all child and parent measures were dichotomised into two categories (within normal range or above cut-off indicating risk) based on evidence-based or country-specific thresholds. This approach allowed for consistent interpretation of associations between child difficulties and parental wellbeing across diverse cultural and methodological contexts. A summary of relevant standardised instruments used to assess the four domains of parental QoL, parental stress, child’s emotional/behavioural difficulties and adaptive and cognitive functioning are reported in **Table 1**.

**Table 1:** Measures used across different countries.

Measures	Australia	Singapore	UK	Romania	Hungary
Emotional/behavioural differences	CBCL	SDQ	SDQ	SMFQ	SDQ
Cognitive/adaptive differences	VABS	VABS	VABS	VABS	FSIQ
Parental stress	PSI	PSI	PSS	PSS	PSS
Quality of life	QoLA	QoLA	QoLA	–	QoLA
Sociodemographic and clinical indicators	Child’s age, gender, co-occurring conditions, parent’s age, gender, and education level.	Child’s age, gender, co-occurring conditions, parent’s age, gender, and education level.	Child’s age and gender.	Child’s age, gender, co-occurring conditions, parent’s age, and gender.	Child’s age, gender, co-occurring conditions, parent’s age, gender, and education level.

CBCL, Child Behavioural Checklist; SDQ, Strengths and difficulties questionnaire; SMFQ, Short Mood and Feelings Questionnaire; VABS, Vineland Adaptive Behaviour Scales; FSIQ, Full-Scale IQ; PSS, Parental Stress Scale; PSI, Parental Stress Index; QoLA, Quality of Life in Autism.

#### 2.3.1 Parental wellbeing outcomes

Parental QoL was assessed using the Quality of Life in Autism (QoLA), a self-reported questionnaire which includes two components – Part A (28 questions) assessing the overall parental QoL, and Part B (20 questions) assessing the impact of child’s autistic features on parents (22). Scores for Part A range from 28 to 140 while Part B scores range from 20 to 100. Higher scores reflect better QoL (part A) and less impact of child autism features on parents (part B). The English version of the QoLA was used in the UK, Australia, and Singapore, while the Hungarian version was used in Hungary (23). The QoLA scale has demonstrated good internal consistency and construct validity in prior studies (22, 24, 25). In this sample, there was excellent internal consistency with Cronbach’s alpha ranging from 0.95 (Part A) to 0.97 (Part B). For the purpose of this study, scores were dichotomised using one standard deviation below country specific sample mean as a cut-off, with scores below this threshold indicating poorer QoL or greater impact of autistic features. Data on parental QoL were available from four countries: Australia, Hungary, Singapore, and the UK.

*Parental Stress* was measured using the Parental Stress Scale (PSS) in Hungary, UK, and Romania, whereas the Parenting Stress Index (PSI) was used in Australia and Singapore. The PSS is an 18-item self-report questionnaire assessing factors contributing to and relieving parenting stress, with scores ranging from 18 (lowest stress) to 90 (highest stress) (26). Given there are no clinical cut offs for the PSS, and interpretation of results depends on the context of each individual (27), scores above one standard deviation above the country specific sample median were categorised as high stress. The PSI is a 120-item questionnaire covering both child-related (e.g., behaviour, demands) and parent-related (e.g., role restriction, social isolation) domains, with scores above the 90^th^ percentile indicating clinically significant stress (28). In this sample, there was excellent internal consistency with Cronbach’s alpha of 0.90 for PSS and 0.81 for PSI scales.

#### 2.3.2 Child’s autistic traits

Emotional/behavioural difficulties were assessed using parent-reported Strengths and Difficulties Questionnaire (SDQ) (used in Hungary, the UK, and Singapore), Short Mood and Feelings Questionnaire (SMFQ) (used in Romania), and Child Behaviour Checklist (CBCL) (used in Australia). The SDQ measures five domains of emotional, conduct, hyperactivity/inattention, peer relationship problems, and prosocial behaviour (29). Scores range from 0 to 40, with higher scores indicating greater difficulties. For analysis, children scoring 20 to 40 (very high) were classified as having behavioural and emotional difficulties (29). The SDQ has been linked to clinical diagnoses of behavioural and emotional disorders and has strong psychometric properties across cultures (30, 31). The SMFQ is a 13-item questionnaire validated as a screening tool for depression in children over six years (32). Scores range from 0 to 26, with higher scores indicating higher risk of depressive symptoms; scores≥8 suggest elevated risk. The CBCL is a 113-item parent-report questionnaire assessing behavioural and social problems in children aged 6–18 years (33, 34). Scores are converted to T-scores adjusted for age and sex, with scores ≥70 indicating clinical significance. For the purpose of this study, the total CBCL scores which aggregates internalising (e.g., anxiety, mood, withdrawal) and externalising (e.g., aggression, rule-breaking) behaviours was dichotomised as within normal range and at-risk. In this sample, there was good to excellent internal consistency with Cronbach’s alpha of 0.89 for SMFQ, 0.78 for SDQ, and 0.94 for CBCL.

Adaptive and cognitive functioning was assessed using the Vineland Adaptive Behaviour Scales, Second Edition (VABS-2) and the Wechsler Intelligence Scale for Children, Fourth Edition (WISC-IV). VABS-2 was used in Australia, UK, Singapore, and Romania whereas the WISC-IV was used in Hungary. The VABS-2 assessment investigates the adaptive functioning across domains of communication, daily living, and socialisation (35, 36). The interview edition, validated for children from birth to 18 years 11 months, was used in this study. Scores are standardised with a mean of 100 and a standard deviation of 15, with scores below 85 indicating lower-than-average adaptive functioning. The VABS-2 has demonstrated robust validity in children with autism and other developmental conditions. The WISC-IV assessment measures cognitive functioning across four domains: verbal comprehension, perceptual reasoning, working memory, and processing speed, with the Full-Scale IQ (FSIQ) reflecting overall cognitive ability (37, 38). Scores are standardised with a mean of 100 and a standard deviation of 15, with scores below 85 indicating below-average cognitive functioning. In this sample, there was good to excellent internal consistency with Cronbach’s alpha of 0.86 for VABS-2. Internal consistency was not computed for the WISC-IV index domains as each represents a single standardised composite score rather than a multi-item scale.

### 2.4 Statistical analysis

Descriptive statistics such as means, and standard deviations were computed for continuous variables whereas counts with percentages were calculated for categorical variables. Primary analysis included multivariable logistic regression models to examine associations between autistic child’s difficulties (emotional/behavioural, cognitive/adaptive) and parental outcomes (stress and QoL) while adjusting for covariates (child’s age and sex). Findings were reported as adjusted odds ratios (AORs) and 95% confidence intervals (CIs), and statistical significance was defined as p<0.05. Additionally, to account for differences across countries and measurement tools, models included random effects that allowed for variation in outcomes between countries and the different standardised measures used by different countries. The proportion of variation explained by the country- and measure-level differences was estimated using intraclass correlation coefficients (ICCs).

Missing data were handled using available-case analysis for each regression model. Participants with missing values for variables included in a given model were excluded from that analysis only. Given the minimal set of harmonised variables available in this pooled multinational dataset, multiple imputation was not feasible. Analyses were conducted using R (version 4.3) with the *lme4* package for multilevel modelling.

## 3 Results

### 3.1 Sample characteristics

A total of 219 families participated across the five countries: Australia (n=82), Singapore (n=30), UK (n=37), Romania (n=30), and Hungary (n=40). The characteristics of the sample by country are presented in **Table 2**. Across the pooled sample, children ranged from early childhood (preschool sample in Australia) to adolescence (UK), allowing comparisons across different developmental stages. Overall, patterns of emotional/behavioural and cognitive/adaptive functioning varied substantially across the countries. In particular, the prevalence of emotional and behavioural difficulties was highest among children in the UK (75.7%), followed by Romania (43.3%) and Australia (31.7%), while Singapore recorded no children classified in the ‘at risk’ clinical range. Additionally, cognitive/adaptive difficulties were most prevalent in the Australia (98.3%), UK (94.3%), and Singapore (88.2%) whereas Hungary (5.3%) reported markedly lower rates. Levels of parental wellbeing also differed by country with the highest proportion of parental stress scores in Australia (61.4%) and Singapore (46.7%). On the contrary, parental QoL showed less variability across the countries.

**Table 2:** Descriptive summary of participants by country.

Characteristics	Australia N = 82 n (%)	Singapore N = 30 n (%)	UK N = 37 n (%)	Romania N = 30 n (%)	Hungary N = 40 n (%)
Child’s age in years, mean (SD)	4.04 (0.56)	5.60 (1.16)	15.32 (3.40)	13.03 (3.03)	12.40 (3.14)
Missing	0 (0.00)	0 (0.00)	0 (0.00)	0 (0.00)	0 (0.00)
Child’s gender					
*Male*	70 (85.37)	25 (83.33)	37 (100.00)	20 (86.96)	32 (80.00)
*Female*	12 (14.63)	5 (16.67)	0 (0.00)	3 (13.04)	8 (20.00)
*Missing*	0 (0.00)	0 (0.00)	0 (0.00)	7 (23.33)	0 (0.00)
Co-occurring conditions					
*No*	45 (54.9)	10 (33.3)	NA	14 (46.7)	29 (72.5)
*Yes*	37 (45.1)	19 (63.3)	NA	16 (53.3)	11 (27.5)
*Missing*	0 (0.00)	1 (3.3)	NA	0 (0.00)	0 (0.00)
Parent’s age in years, mean (SD)	29.7 (4.9)	38.7 (5.7)	NA	41.9 (5.5)	44.9 (5.2)
*Missing*	0 (0.00)	0 (0.00)	NA	0 (0.00)	0 (0.00)
Parent’s gender					
*Male*	1 (1.2)	8 (26.7)	NA	0 (0.00)	12 (30.0)
*Female*	55 (67.1)	22 (73.3)	NA	30 (100.0)	28 (70.0)
*Missing*	26 (31.7)	0 (0.00)	NA	0 (0.00)	0 (0.00)
Education level					
*Up to secondary school education (high school or equivalent)*	18 (22.0)	6 (20.0)	NA	NA	3 (7.5)
*Post-secondary education (vocational training, college, university, or higher)*	59 (72.0)	24 (80.0)	NA	NA	37 (92.5)
*Missing*	5 (6.1)	0 (0.00)	NA	NA	0 (0.00)
Emotional/behavioural differences					
*Not at risk*	56 (68.29)	30 (100.00)	9 (24.32)	17 (56.67)	29 (72.50)
*At risk*	26 (31.71)	0 (0.00)	28 (75.68)	13 (43.33)	11 (27.50)
*Missing*	0 (0.00)	0 (0.00)	0 (0.00)	0 (0.00)	0 (0.00)
Cognitive/adaptive differences					
*Not at risk*	1 (1.69)	2 (11.76)	2 (5.71)	8 (32.00)	36 (94.74)
*At risk*	58 (98.31)	15 (88.24)	33 (94.29)	17 (68.00)	2 (5.26)
*Missing*	23 (28.05)	13 (43.33)	2 (5.41)	5 (16.67)	2 (5.00)
Parental stress					
*Not at risk*	22 (38.60)	16 (53.33)	28 (87.50)	24 (80.00)	33 (82.50)
*At risk*	35 (61.40)	14 (46.67)	4 (12.50)	6 (20.00)	7 (17.50)
*Missing*	25 (30.49)	0 (0.00)	5 (13.51)	0 (0.00)	0 (0.00)
Quality of life Part A					
*Not at risk*	45 (76.27)	25 (83.33)	20 (86.96)	NA	34 (85.00)
*At risk*	14 (23.73)	5 (16.67)	3 (13.04)	NA	6 (15.00)
*Missing*	23 (28.05)	0 (0.00)	14 (37.84)	30 (100.00)	0 (0.00)
Quality of life Part B					
*Not at risk*	45 (78.95)	22 (73.33)	20 (86.96)	NA	10 (76.92)
*At risk*	12 (21.05)	8 (26.67)	3 (13.04)	NA	3 (23.08)
*Missing*	25 (30.49)	0 (0.00)	14 (37.84)	30 (100.00)	27 (67.50)

NA, Not available.

### 3.2 Findings of the regression analyses

Results of the multivariable binary logistic regression analyses examining the associations between child-level predictors (emotional/behavioural and cognitive/adaptive difficulties) and parental wellbeing outcomes (QoLA Part A, QoLA Part B, and Parental Stress), adjusting for country, measurement, child’s age, and sex are presented in **Table 3**.

**Table 3:** Associations between child emotional/behavioural and cognitive/adaptive differences with parental quality of life and parental stress across countries and measures.

Predictors	AOR (95% CI)	Countries included	Country level variance	Country ICC	Measures level variance	Measure ICC
Outcome variable – QoLA Part A						
Emotional/behavioural differences		Australia, Hungary, Singapore, UK				
*Not at risk*	Reference					
*At risk*	0.36 (0.11, 0.97)*		0.00	0.00	0.00	0.00
Cognitive/adaptive differences						
*Not at risk*	Reference					
*At risk*	1.05 (0.32, 3.49)		0.00	0.00	0.00	0.00
Outcome variable – QoLA Part B						
Emotional/behavioural differences		Australia, Hungary, Singapore, UK				
*Not at risk*	Reference					
*At risk*	0.43 (0.13, 1.39)		0.00	0.00	0.00	0.00
Cognitive/adaptive differences						
*Not at risk*	Reference					
*At risk*	0.52 (0.15, 1.81)		0.00	0.00	0.00	0.00
Outcome variable – Parental stress						
Emotional/behavioural differences		Australia, Romania, Hungary, Singapore, UK				
*Not at risk*	Reference					
*At risk*	2.11 (1.08, 4.54)*		0.00	0.00	0.00	0.00
Cognitive/adaptive differences						
*Not at risk*	Reference					
*At risk*	1.29 (0.52, 3.19)		0.00	0.00	0.00	0.00

AOR, Adjusted odds ratio – adjusted for child’s age and sex; ICC, intraclass correlation coefficient; *p-value<0.05.

Parents of children with emotional and behavioural difficulties reported significantly lower quality of life on QoLA Part A (AOR = 0.36, 95% CI 0.11–0.97) and twice higher odds of experiencing stress (AOR = 2.11, 95% CI 1.08-4.54). These findings indicate that persistent emotional and behavioural challenges are linked to substantial strain on parental wellbeing and family functioning. In contrast, cognitive and adaptive difficulties were not significantly associated with parental outcomes but showed trends in expected directions, reflecting the intensive demands of early caregiving.

In terms of sociodemographic factors, increased child age was significantly associated with lower odds of parental stress (AOR = 0.81, 95% CI 0.74–0.88), but was not a significant predictor of QoLA Part A or Part B outcomes. Further analyses examining interaction effects between child age and emotional/behavioural difficulties, as well as cognitive/adaptive difficulties, indicated that none of these interactions were statistically significant.

Between-country and measure-level variance were negligible across all outcomes (ICC = 0.00), indicating minimal clustering by country or assessment instrument and supporting the validity of cross-country comparisons.

## 4 Discussion

### 4.1 Summary of findings

This multinational study represents one of the early attempts to harmonise parent- and child-level autism-related wellbeing data across five culturally and geographically diverse countries using pooled analytical approaches to examine the associations between children’s emotional/behavioural and cognitive/adaptive difficulties and parental wellbeing through a transcultural perspective. Given the substantial challenges in cross-cultural autism research, including differences in healthcare systems, assessment practices, language adaptations, and locally available datasets, this study represents an important early step toward developing harmonised international evidence on parental wellbeing in autism. The findings of this study indicate that emotional and behavioural issues in autistic children were found to be strongly associated with reduced QoL and elevated stress in parents, highlighting the sustained impact of persistent behavioural challenges on family functioning. Additionally, cognitive and adaptive difficulties were not significantly associated with parental stress but showed trends toward lower QoL and higher stress, suggesting that early caregiving demands contribute to parental strain. Importantly, between-country and measure-level variance was negligible across all parental wellbeing outcomes, indicating that these associations were consistent across countries and assessment tools.

### 4.2 Emotional and behavioural difficulties and parental wellbeing

Findings of our study showed that emotional and behavioural difficulties in autistic children were significantly associated with poor parental QoL and elevated stress (39, 40). These findings are consistent with prior studies that have shown that such issues, particularly externalising and internalising behaviours often necessitate continual and intensive caregiving (39–41). For example, externalising behaviours such as aggression, hyperactivity, and tantrums can disrupt daily routines, heighten safety concerns, and require parents to maintain constant vigilance, resulting in chronic stress and poor wellbeing (41, 42). Similarly, internalising behaviours such as anxiety, withdrawal, and emotional dysregulation can be also necessitate parents to seek and provide ongoing additional support including navigating complex mental health needs (42). Further, if left untreated, these difficulties compound over time across childhood and adolescence, thereby intensifying the ongoing caregiving load and contributing to sustained negative wellbeing (43). Therefore, early screening programs can enable early intervention of emotional and behavioural difficulties in autistic children, which potentially results in better health and wellbeing outcomes for both children and parents (44).

### 4.3 Cognitive and adaptive difficulties and parental QoL

Although cognitive and adaptive difficulties were not significantly associated with parental wellbeing outcomes in this study, the observed trends toward lower parental quality of life and higher stress are consistent with previous research. Children with greater cognitive challenges or lower adaptive functioning may require more intensive parental support for daily activities, learning, and behavioural regulation (8, 9). Difficulties with self-care, transitions, and social interactions may increase the demands placed on parents, particularly during early developmental stages when children require greater hands-on support (8, 9). Therefore, early identification and interventions aimed at strengthening adaptive skills and promoting functional independence may not only improve child outcomes but also enhance parental wellbeing.

### 4.4 Relationship between child's age and parental wellbeing outcomes

Child’s age was found to be a modest but significant predictor, with increased age associated with lower odds of parental stress, although it was not related to parental QoL outcomes. This pattern is broadly consistent with literature suggesting changes in caregiver burden across developmental stages (45, 46). However, in contrast to some prior studies that report age-related variation in the strength of associations between child symptom severity and parental wellbeing (46), our interaction analyses indicated that the relationships between child-level difficulties and parental outcomes were stable across the observed age range. This suggests that while overall stress levels may decrease with child age, the relative impact of emotional/behavioural and cognitive/adaptive difficulties on parental wellbeing remains consistent across developmental stages (47).

### 4.5 Cross-country and cultural considerations

Our findings suggest that the relationship between child’s autistic differences and parental wellbeing is consistent across countries, as evidenced by the negligible between-country variance. This indicates that the challenges associated with raising an autistic child with additional support needs is universal and appears to affect parents in similar ways, regardless of sociocultural norms, parenting practices, or healthcare systems across countries (21). Further, the negligible measure-level variance indicates that, although different countries employed different standardised instruments to assess parental wellbeing and child difficulties, the observed associations remained consistent across measures. The consistency may partly reflect the cultural adaptation and validation of these instruments within their respective contexts, which likely enhances their ability to measure similar underlying constructs across diverse populations (48, 49). Consequently, the findings strengthen the robustness and generalisability of the results, indicating that the observed relationships are unlikely to be artefacts of specific measurement tools but rather reflect stable patterns across heterogeneous yet validated measures. However, cross-cultural comparisons should be interpreted with caution given the use of different assessment instruments across countries and variation in the sampling and age range of children and adolescents included. Although harmonisation procedures and multilevel modelling were applied to reduce methodological heterogeneity, residual differences in measurement and developmental stage may still influence comparability and the extent to which observed patterns reflect true cross-cultural equivalence.

At the same time, variability in the levels of autistic child’s features and parental wellbeing outcomes observed in descriptive sample characteristics across countries emphasises the importance of considering contextual factors in shaping parental wellbeing. Evidence from Asian contexts suggests that parental stress may be heightened by cultural expectations of parental responsibility, stigma, and limited access to formal autism services, which can discourage help-seeking despite the presence of extended family networks (50–53). In Western countries, although there is availability of relatively robust healthcare systems, early intervention services, and advocacy networks, parents may still experience substantial stress related to navigating complex and fragmented service systems, long waiting times, high out-of-pocket costs, and cultural norms that emphasise parental autonomy and advocacy (54). Additionally, more nuclear family structures common in Western societies may reduce informal caregiving support, further contributing to parental stress (55). Central and Eastern European countries appear intermediate, where delayed diagnosis, persistent stigma, and systemic constraints in care and education systems shape parental stress (23, 56). For example, parental satisfaction with their child’s educational situation has been shown to directly influence QoL in Hungary (23). Overall, these findings emphasise the need for universal strategies for supporting a child’s emotional and behavioural issues but also the need for culturally appropriate supports for parental wellbeing.

### 4.6 Strengths and limitations

A key strength of this study is the use of multinational data enabling examination of the associations between child’s autistic differences and parental wellbeing outcomes across different countries with diverse cultural contexts. Whilst methodological heterogeneity is an inherent challenge in multinational research collaborations, the ability to identify consistent associations despite these differences may strengthen the broader relevance of the findings. However, several limitations should be considered. First, the cross-sectional design means that causal relationships between child’s autistic differences and parental wellbeing cannot be determined. Second, reliance on parent-reported measures may introduce reporting bias. Thirdly, several potentially important child- and parent-level variables, including but not limited to, age of autism diagnosis, autism severity, co-occurring psychiatric, or treatment status were not collected across all participating datasets and therefore could not be included in the pooled analyses. The absence of these harmonised variables may have limited the ability to account for factors influencing parental wellbeing outcomes across countries. In addition, parental QoL data were unavailable from Romania, which limited the completeness of cross-country comparisons for one of the principal study outcomes and may have reduced the representativeness of pooled QoL analyses. Furthermore, the unequal sample sizes across participating countries may have influenced cross-country comparisons and reduced the precision and stability of estimates for countries with smaller sample sizes. Finally, although descriptive statistics revealed significant variations in parental stress and quality of life across countries, these differences were not fully reflected in the regression models. This may be attributed to limited sample sizes, missing data, measurement variability, or statistical adjustments that account for multiple covariates, which may have affected statistical power and comparability. Future research should prioritise larger, cross-country datasets with harmonised assessment protocols and sampling, strengthening the cross-cultural comparability and generalisability of findings.

### 4.7 Implications for practice and policy

The findings of this study highlight the need for early identification and timely support for autistic children through tiered models of care that are tailored to the child’s level and profile of emotional, behavioural, cognitive, and adaptive needs. Interventions should adopt a family-centred approach that recognises the interconnected wellbeing of children and parents. In addition to child-focused supports, services should incorporate strategies to strengthen parental wellbeing, including psychoeducation, mental health support, and peer networks in a flexible format (in-person and/or digital). Emerging literature including a recent study from Singapore illustrates how parents value mental health support for themselves but find it hard to access this amidst competing child-related demands (57). At a policy level, these findings highlight the importance of improving the availability, accessibility, and coordination of healthcare, educational, and social services, for both children and their parents, alongside financial and respite supports, to promote equitable support for parental wellbeing.

## 5 Conclusion

This multinational study showed that emotional and behavioural difficulties in autistic children are strongly associated with reduced parental quality of life and elevated stress, whereas cognitive and adaptive difficulties showed weaker, non-significant associations with parental wellbeing. The lack of country and measure-level variance suggests that these relationships are consistent across diverse cultural contexts and robust across validated assessment tools. Despite methodological and contextual heterogeneity, these findings demonstrate the feasibility and value of harmonising multinational autism datasets to better understand parental wellbeing across diverse cultural settings. Collectively, these results highlight the urgent need for early identification and tailored interventions and supports for children with emotional, behavioural, and cognitive needs, as well as the importance of culturally informed, family-centred interventions that promote parental wellbeing.

## Data Availability

The raw data supporting the conclusions of this article will be made available by the authors, without undue reservation.
