# Correction: Cross-cultural associations between behavioural, emotional, and cognitive differences in autistic children and parental wellbeing: evidence from five countries

**DOI:** 10.3389/fpsyt.2026.1951636

**Published:** 2026-08-06

**Authors:** James Rufus John, William Suwandi Budiman, Christa Lam-Cassettari, Poppy Zenzi Grimes, Kiran Hingorani, Ying Qi Kang, Ramkumar Aishworiya, Erdembileg Sundarimaa, Marta Volgyesi-Molnar, Krisztina Stefanik, Agota Szekeres, Ileana Mardare, Florina Rad, Valsamma Eapen

**Affiliations:** 1 School of Clinical Medicine, University of New South Wales, Sydney, NSW, Australia; 2 Academic Unit of Infant, Child, and Adolescent Psychiatry Services, South Western Sydney Local Health District, Liverpool, NSW, Australia; 3 Ingham Institute of Applied Medical Research, Liverpool, NSW, Australia; 4 Swalcliffe Park School CIO, Oxfordshire, United Kingdom; 5 Yong Loo Lin School of Medicine, National University of Singapore, Singapore, Singapore; 6 Child Development Unit, Khoo Teck Puat – National University Children’s Medical Institute, National University Hospital, Singapore, Singapore; 7 Hungarian Academy of Sciences - ELTE University ‘Autism in Education’ Research Group, Budapest, Hungary; 8 Faculty of Special Education, Institute of Special Needs Education for People with Atypical Behaviour and Cognition, ELTE University, Budapest, Hungary; 9 Hungarian University of Agriculture and Life Sciences, Institute of Education, Kaposvár, Hungary; 10 University of Medicine and Pharmacy “Carol Davila”, Bucharest, Romania

**Keywords:** autism spectrum disorder, autistic difficulties, parental stress, quality of life, transcultural

Affiliation 9, “University of Medicine and Pharmacy “Carol Davila”, Bucharest, Romania” was erroneously assigned to author Agota Szekeres. Additionally, Agota Szekeres’s affiliation “Hungarian University of Agriculture and Life Sciences, Institute of Education, Kaposvár, Hungary**”** was omitted.

To ensure all author affiliations are listed sequentially, affiliation 9 has been updated to “Hungarian University of Agriculture and Life Sciences, Institute of Education, Kaposvár, Hungary” (assigned to Agota Szekere) and affiliation 10 has been updated to “University of Medicine and Pharmacy “Carol Davila”, Bucharest, Romania” (assigned to Ileana Mardare and Florina Rad).

The original version of this article has been updated.

